# Professional identity formation among undergraduate pre-medical students: a scoping review protocol

**DOI:** 10.1186/s13643-023-02329-8

**Published:** 2023-09-23

**Authors:** Meklit Endalcachew, Jennifer Deberg, Melissa Swee, Manish Suneja, Bharat Kumar

**Affiliations:** 1 College of Liberal Arts and Sciences, University of Iowa, University of Iowa, Iowa City, IA USA; 2 University of Iowa Hardin Library, University of Iowa, Iowa City, IA USA; 3 Department of Internal Medicine, University of Iowa Carver College of Medicine, Iowa City, IA USA; 4 Division of Nephrology, University of Iowa Carver College of Medicine, Iowa City, IA USA; 5 Division of Immunology, University of Iowa Carver College of Medicine, Iowa City, IA USA

## Abstract

**Background:**

Professional Identity formation is the process by which learners internalize a profession’s values, behaviors, and perceptions. With respect to physicians, this occurs at multiple levels of medical education, including the undergraduate, graduate, and continuing medical education stages. Professional identity formation likely starts even earlier, during the undergraduate pre-medical years but, to date, no known scoping or systematic review has been conducted on this topic. The objective of this scoping review is to systematically map the literature on professional identity formation among undergraduate pre-medical students.

**Methods:**

This review protocol has been designed following the Arksey and O’Malley framework. We will search MEDLINE, CINAHL, Embase, and Scopus, as well as relevant grey literature, conference proceedings, and citations of selected articles. Inclusion criteria are articles (1) written in the English language, (2) involving undergraduate pre-medical students in the USA and Canada, and (3) containing original data about professional identity formation. Two independent reviewers will evaluate the titles, abstracts, and full articles for eligibility. A third reviewer will help resolve any disputes. Once the full text of articles are obtained, data will be abstracted using a standardized form. A narrative summary of findings will then be conducted, as well as a consultation exercise with university pre-medical students, pre-med advisors, and first-year medical students.

**Discussion:**

By conducting this scoping review, we expect to gain a better understanding of how the experiences of undergraduate pre-medical students impact their professional identity formation. These findings will help to identify gaps in the literature, to better characterize professional identity formation in the specific context of the undergraduate pre-medical track, and to outline potential approaches to facilitate professional identity formation among undergraduate pre-medical students.

**Systematic review registration:**

The protocol is registered with the Open Science Framework (htps://osf.io/nfzxc).

**Supplementary Information:**

The online version contains supplementary material available at 10.1186/s13643-023-02329-8.

## Background

Professional identity formation (PIF) is the process of internalizing a profession’s values, behaviors, and perceptions. It is considered one of the most important goals of medical education and occurs throughout undergraduate, graduate, and continuing medical education [1]. In recent decades, there has been increasing recognition that professional identity is a complex construct that is impacted by external contexts, instruction, and reinforcement, rather than a product of unchanging and inherent qualities of an individual’s personality or character [2]. The publication of the Educating Physicians: A Call for Reform of Medical School and Residency, by the Carnegie Foundation, in 2010 has championed the role of the PIF framework in physicians-in-training and catalyzed further research on the topic [3]. While research has been conducted and compiled on professional identity formation in undergraduate medical school and residency programs, little is known about how experiences of undergraduate pre-medical students impacts professional identity formation [4–8].

In the USA and Canada, most undergraduate students pursuing further studies in medical school enter the pre-medical (pre-med) educational track [9]. Although not entirely standardized, the pre-med track involves not only formal coursework but also extracurricular activities, research, and the process of applying to medical schools [10]. Collectively, these pre-med experiences not only provide training for further study but also expose individuals to the core values and beliefs upheld by the medical profession. Therefore, some form of professional identity formation occurs during this time, even before the commitment of starting formal medical training. Characterizing what is known in peer-reviewed literature is an important step towards understanding the early steps of professional identify formation and to identify steps that can be taken to help support future physicians as they start on their journey towards their careers. Additionally, describing the needs of various subpopulations and the evolution of this professional identity formation may empower further innovations and research within the field of medical education.

## Methods

### Objectives

This scoping review protocol aims to identify and synthesize the literature on professional identity formation among undergraduate students planning to enter medical school. The PS (Population, Situation) tool [11] has been used to guide the scope of our literature review:

1. Population: undergraduate medical students in the USA and Canada
2. Situation: professional identity formation within the premedical curriculum

Because identity is an expansive concept and there do not exist prior literature reviews about professional identity formation among undergraduate pre-medical students, a scoping review synthesizing the state of the current knowledge and mapping areas for future investigations is most appropriate [12]. We will limit our review to medical students in the USA and Canada to homogenize the population studied, since undergraduate pre-medical curricula vary considerably among different countries and many countries allow entry into medical school with after obtaining a high school degree without a university degree.

### Methodological framework

This review protocol has been registered within the Open Science Framework database (registration: https://osf.io/nfzxc). The investigators have used the Preferred Reporting Items for Systematic Reviews and Meta-Analyses (PRISMA) extension for scoping reviews (PRISMA-ScR) to guide and document our approach in anticipation for future publication of results (Supplement 1) [13]. Additionally, this protocol has been reported in accordance with the reporting guidance provided in the PRISMA extension for protocols (PRISMA-P) (Supplement 2) [14].

Arksey and O’Malley’s five step framework has been used to plan our methodological approach [15]. Arksey and O’Malley outline five steps as part of the scoping review process: (1) identification of the research question, identification of relevant studies, selection of relevant studies, charting of findings, and the summarization and reporting of results. This framework is particularly suitable because it balances the need for rigor and transparency in searching the literature while maintaining an iterative process in order to identify gaps in existing literature. Additionally, in line with more recent practices and to ensure learner-centeredness of our review, we have included a sixth step of stakeholder analysis [16].

### Stage 1: identifying the research question

To better understand the contextual issues related to professional identity formation among undergraduate pre-medical students, we identified the following questions:

1. How do undergraduate pre-medical students view their current and future professional identities?
2. What are the contextual factors that affect or influence professional identity formation among undergraduate pre-medical students?
3. How does professional identity formation among undergraduate pre-medical students influence professional identity formation during subsequent stages of career development?
4. What theoretic frameworks and paradigms regarding professional identity formation have been developed with respect to undergraduate pre-medical students?
5. What types of strategies or interventions to foster professional identity formation among undergraduate pre-medical students are available in literature?

### Stage 2: identifying relevant studies

The search will be conducted for published and unpublished (grey) literature. The following electronic databases will be queried: MEDLINE (11/23/1963 –7/1/2023, accessed through https://pubmed.ncbi.nlm.nih.gov/), CINAHL (1/1/1981–7/1/2023, accessed through https://purl.lib.uiowa.edu/ebsco/cinahl), Embase (1/1/1974–7/1/2023 accessed through https://www-embase-com.proxy.lib.uiowa.edu/), and Scopus (1/1/1966–7/1/2023, accessed through https://www-scopus-com.proxy.lib.uiowa.edu/). Medical Subject Headings (MeSH) terms and headings have been identified, based on prior studies (Supplement 3). Broadly, the search is composed of two components: (1) “professional identity” and (2) “pre-medical students.” The ‘AND’ Boolean operator is used to maximize the potential to identify articles that relate to both of these concepts. Both standardized terms (depending on the electronic database) and relevant free-text terms have been used in order to facilitate the literature search.

The search strategy will be piloted to check the appropriateness of keywords and databases. Keywords may be refined to include specific domains of professional identity formation, including professionalism, psychosocial identity development, and formation.

To find additional studies, a hand search of references of included studies will be conducted. Additionally, we will search dissertations/theses on ProQuest Dissertations and Theses and conference abstracts (Embase Conference Abstracts).

Potential relevant grey literature will be identified through targeted searches of dissertations/theses (ProQuest Dissertations & Theses Global), conference abstracts (EMBASE Conference Abstracts, Conference Proceedings Citation Index—Science and Social Science and Humanities), and other grey literature databases (Grey Literature Report, OpenGrey, Web of Science Conference Proceedings).

### Stage 3: study selection

#### Inclusion and exclusion criteria

Once duplicate results are identified and removed, title and abstract screening will be guided by the research questions. Both qualitative and quantitative studies will be considered for inclusion, so long as they focus on professional identity formation among undergraduate students planning to enter medical school. These include retrospective and prospective investigations with original data. We will also conduct a hand-search of the reference lists of included studies to identify additional studies of relevance, as well as the reference lists of any review articles, commentaries, and perspectives that may not contain original data from studies.

We will use the following exclusion criteria to guide the selection of students. Firstly, research that does not focus on the undergraduate population within the USA or Canada will be excluded. Secondly, manuscripts that focus on professional identity formation during the undergraduate years for other health professions will also be excluded. Thirdly, only articles written in the English language will be considered.

#### Full-text screening

All eligible articles will be screened through and categorized in Endnote X9 (Clarivate®; Philadelphia, PA). Two authors (BK and ME) will independently screen title and abstracts of all eligible articles to determine whether the study should be included in the review. The full text of selected articles will then be obtained through the University of Iowa Library System. Two authors (BK and ME) will independently conduct full-text screening of the selected studies. A third reviewer (MLS) will adjudicate any significant discrepancies that cannot be resolved, through discussion and consensus. The inter-rater Kappa agreement between reviewers will be calculated.

### Stage 4: charting the data

Two reviewers (MS and MLS) will have developed a preliminary data extraction sheet using Microsoft Excel (Microsoft®; Redmond, WA) with which to compile data from articles meeting the above criteria. Information from the text, tables, figures, and supplementary material will all be considered sources of data. These include: (1) title of the study, (2) publication, (3) aim of the study, (4) study setting (e.g., location and dates of inquiry), (5) study population (e.g., year of undergraduate education), (6) sampling method, (7) study design, (8) contextual factors affecting professional identity formation, (9) theoretical framework or paradigm, (10) strategies or interventions to foster professional identity formation, (11) data collection methods, (12) data analysis, (13) conclusion, and (14) outcomes. An additional field of free-text comments will also be included. Cells of any missing data will be kept unfilled. In the case of discrepancies, a third reviewer (BK) will resolve the situation through discussion and adjudication. Because scoping reviews seek to map the landscape, no risk of bias assessment will be conducted.

### Stage 5: collecting, summarizing, and reporting results

**Fig. 1:**
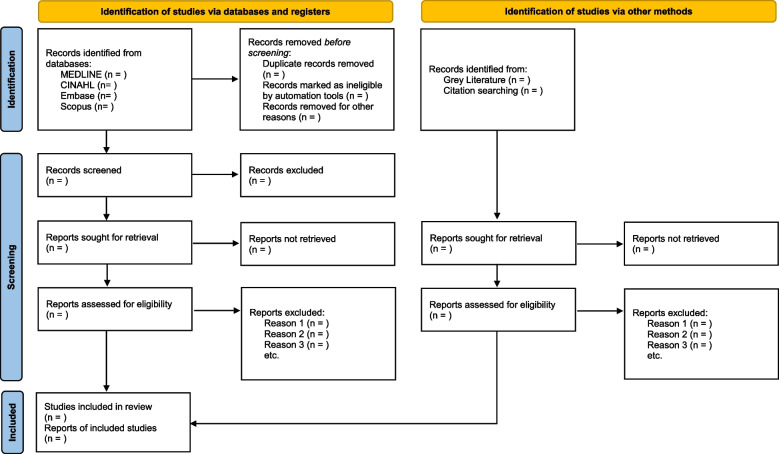
PRISMA 2020 flow diagram for new systematic reviews which included searches of databases, registers and other sources

The PRISMA guidelines will be used to report and summarize the results of the literature search (Fig. 1). We anticipate a wide variety of studies, and so will provide descriptive accounts of the concepts emerging from our literature search that would preclude a meta-synthesis. We will then synthesize study findings based on themes emerging from extracted data, in line with the five research questions. Additionally, studies that address research questions 1–4 will be classified based on Kegan’s framework on identity formation and Cruess’s schematic representation of professional identity formation and socialization of medical students and residents [17].

Kegan’s framework describes a six-stage helical pathway in which perspectives, values, emotional control, and reflection contribute to the process of the development of personal relationships with others and to society. Kegan’s framework has been used previously to explain the developmental process among medical trainees. Cruess’s schematic representation builds off of Kegan’s stages and provides a schematic representation for professional identity formation and socialization among medical trainees. Due to their relative conceptual simplicity, their previous use in medical education, and their complementary approaches, applying both Kegan’s framework and Cruess’s schematic representation seem to be the most appropriate method to summarizing and reporting professional identity formation among undergraduate students planning to enter medical school [18].

As part of reporting the results, we will use Microsoft Excel (Microsoft®; Redmond, WA) to tabulate how frequently each concept is discussed. The results of these calculations will be the basis of a table and/or a figure to summarize the data.

### Stage 6: consultation exercise

We will consult with stakeholders to provide insights beyond what is reported in literature. To ensure that the study is learner-centric, we will consult four undergraduate pre-medical students and two pre-medical advisors as well as two first-year medical students. We will present the results and analyze their responses so that their perspectives can be integrated into the scoping review. A specific focus will be on how these results resonate with their own experiences and what gaps do they see within the literature itself, and what further investigations are needed to close these gaps.

## Discussion

This study constitutes the first step in understanding how undergraduate pre-medical students form their professional identities. This protocol reports a comprehensive methodology in a rigorous and transparent manner. Results will be disseminated through a peer-reviewed publication and through regional, national, and international conferences.

The strengths of this proposed approach include the use of a rigorous methodology (Arksey and O’Malley’s Framework) to search for literature in four large databases. Provisions for identifying grey literature have also been included as part of this scoping review. The addition of a consultation exercise as the sixth step will also allow us to better understand the consequentiality of our results. The major limitation is that is this is a scoping review, rather than a systematic review. While our focus remains on undergraduate students in the USA and Canada interested in pursuing careers as physicians, it remains unclear if the results can be generalizable to other, related groups.

By mapping the landscape regarding professional identity formation among undergraduate pre-medical students, we can identify gaps in knowledge and guide future educational research. Both the methodology and results may benefit other researchers and health professions educators beyond medicine, such as nursing, dentistry, and physical therapy.

### Supplementary Information

**Additional file 1. **Preferred Reporting Items for Systematic reviews and Meta-Analyses extension for Scoping Reviews (PRISMA-ScR) checklist.

**Additional file 2. **PRISMA-P 2015 checklist.

**Additional file 3. **Terms and strategies.

## Data Availability

Data sharing is not applicable to this article because no datasets were generated or analyzed for this study.

## Abbreviations

CINAHL: Cumulative Index to Nursing and Allied Health Literature
MEDLINE: Medical Literature Analysis and Retrieval System Online
MeSH: Medical Subject Headings
PIF: Professional Identity Formation
Pre-Med: Pre-Medical
PRISMA: Preferred Reporting Items for Systematic Reviews and Meta-Analyses
PRISMA-P: Preferred Reporting Items for Systematic Reviews and Meta-Analyses—Protocols
PRISMA-ScR: Preferred Reporting Items for Systematic Reviews and Meta-Analyses – Extension for Scoping Reviews
