# Supplementary material for: Professional identity formation among undergraduate pre-medical students: a scoping review protocol
Source: Syst Rev. 2023 Sep 23;12:171. doi: 10.1186/s13643-023-02329-8 (PMC10517468; doi:10.1186/s13643-023-02329-8)
Supplement: Supplementary file 3 — Additional file 3. Terms and strategies. [file 13643_2023_2329_MOESM3_ESM.docx]

| **Concept: professional identity** | **Concept: pre medical students** |
| --- | --- |
| PubMed MeSH  Professional role  Social identification  Self concept  Socialization  Socialisation  Career choice | PubMed MeSH  Students, premedical  Education, premedical |
| CINAHL Headings  Professional identity  Professional role  Self concept  Social integration  Career planning and development  Socialization  Socialisation  Professionalism | CINAHL headings  Premedical education |
| Embase Emtree terms  Self concept  Social behavior  Professionalism  Scope of practice  Career planning  Socialization  Socialisation | Embase Emtree terms  Premedical student |
| Free text words/phrases  Identity formation  Identity development  Professional identity  Longitudinal identity  Physician identity  Vocational identity  Formation of professional identity  Career maturity  professionalism  Professional development  Career intention (s)  Socialization  Socialisation  Professional role(s)  Social identit (y, ies)  Social identification  Group identification  Group ident (y, ies)  Self perception(s) | Free text words/phrases  Premedical  Pre medical  Pre med  premed  Prospective medical students  Pre professional medical education  Pre professional medical student(s)  Preprofessional medical education  Preprofessional medical student(s) |

**PubMed preliminary 6/25/21**

("Professional Role"[Mesh] OR "Social Identification"[Mesh] OR "Self Concept"[Mesh] OR "Socialization"[Mesh] OR "Career Choice"[Mesh] OR Identity formation[Text Word] OR identity development[Text Word] OR professional identity[Text Word] OR longitudinal identity[Text Word] OR career maturity[Text Word] OR professionalism[Text Word] OR professional development[Text Word] OR career intention[Text Word] OR career intentions[Text Word] OR socialization[Text Word] OR socialization[Text Word] OR professional role[Text Word] OR professional roles[Text Word] OR social identity[Text Word] OR social identities[Text Word] OR social identification[Text Word] OR group identification[Text Word] OR group identity[Text Word] OR group identities[Text Word] OR self perception[Text Word] OR self perceptions[Text Word])

 AND

("Students, Premedical"[Mesh] OR "Education, Premedical"[Mesh] OR Premedical[Text Word] OR pre medical[Text Word] OR prospective medical student[Text Word] OR prospective medical students[Text Word])

**PubMed revised  7/21**

("Professional Role"[Mesh] OR "Social Identification"[Mesh] OR "Self Concept"[Mesh] OR "Socialization"[Mesh] OR "Career Choice"[Mesh] OR Identity [Text Word] OR identification [Text Word] OR career maturity[Text Word] OR professionalism[Text Word] OR professional development[Text Word] OR career intention[Text Word] OR career intentions[Text Word] OR socialization[Text Word] OR socialisation[Text Word] OR professional role[Text Word] OR professional roles[Text Word] OR self perception[Text Word] OR self perceptions[Text Word]) AND ("Students, Premedical"[Mesh] OR "Education, Premedical"[Mesh] OR Premedical[Text Word] OR pre medical[Text Word] OR prospective medical students[Text Word] OR prospective medical students[Text Word] OR premed [Text Word] OR pre med [Text Word] OR pre professional medical education [Text Word] OR pre professional medical student [Text Word] OR pre professional medical students [Text Word])

**CINAHL 7/15/21**

(MH "Professional Role" OR MH "Professional Identity" OR MH "Social Identity" OR MH "Social Integration" OR MH "Self Concept+" OR MH "Socialization" OR MH "Career Planning and Development" OR MH "Professionalism" OR Identit* OR Identification OR "Career maturity" OR Professionalism OR "Professional development" OR "Career intention*" OR "Socialization" OR “Socialisation” OR "Professional role*") AND (MH "Education, Premedical" OR Premedical OR "Pre medical" OR "Prospective medical student*" OR "Pre professional medical" OR “Preprofessional Medical” OR “Pre Med” OR “Premed”)

**Embase**

('professionalism'/exp OR 'scope of practice'/exp OR 'social behavior'/exp OR 'self concept'/exp OR 'career planning'/exp OR 'socialization'/exp OR ‘socialisation’/exp OR identit*:ti,ab,kw OR identification:ti,ab,kw OR 'career maturity':ti,ab,kw OR professionalism:ti,ab,kw OR 'professional development':ti,ab,kw OR 'career intention*':ti,ab,kw OR socialization:ti,ab,kw OR 'professional role*':ti,ab,kw OR 'self perception*':ti,ab,kw) AND ('premedical student'/exp OR premedical:ti,ab,kw OR 'pre medical':ti,ab,kw OR premed:ti,ab,kw OR 'pre med':ti,ab,kw OR 'prospective medical student*':ti,ab,kw OR 'preprofessional medical education':ti,ab,kw OR 'pre professional medical education':ti,ab,kw OR 'preprofessional medical student*':ti,ab,kw OR 'pre professional medical student*':ti,ab,kw)

**Scopus 7/21/21**

( TITLE-ABS-KEY ( identit*  OR  identification  OR  "Career Maturity"  OR  "Professional Development"  OR  professionalism  OR  "Career Intention*"  OR  socialization OR socialisation OR  "Professional Role*"  OR  "Self Perception*" )  AND  TITLE-ABS-KEY ( premedical  OR  "Pre Medical"  OR  premed  OR  "Pre Med"  OR  "Prospective Medical Student*"  OR  "Preprofessional Medical Education"  OR  "Pre Professional Medical Education"  OR  "Pre Professional Medical Student*"  OR  "Preprofessional Medical Student*" ) )
